# Supplementary material for: Remote sensing of salmonid spawning sites in freshwater ecosystems: The potential of low-cost UAV data
Source: PLoS One. 2023 Aug 29;18(8):e0290736. doi: 10.1371/journal.pone.0290736 (PMC10464957; doi:10.1371/journal.pone.0290736)
Supplement: S1 Table — Basic technical specifications of the RGB camera and battery carried by the DJI Mavic Pro and DJI Mavic 2 Zoom drone. (PDF) [file pone.0290736.s001.pdf]

S1 Table. Technical specifications UAV. Basic technical specifications of the RGB camera and battery carried by the DJI Mavic Pro and DJI Mavic 2 Zoom drone.

**Characteristics**	**Specifications DJI Mavic Pro**	**Specifications DJI Mavic 2 Zoom**
Sensor	1/2.3” (CMOS)<br>Effective pixels: 12.35 M	1” CMOS<br>Effective Pixels: 20 million
Lens model	FOV 78.8˚ 26 mm (Format Equivalent: 35 mm)<br>Aperture: f/2.2<br>Distortion <1.5%<br>Focus: 0.5 m to ∞	FOV ca. 77° 35 mm<br>(Format Equivalent: 28 mm)<br>Aperture: f/2.8–f/11<br>Shooting Range: 1 m to ∞
Image size	4000 x 3000 pixels	3992 x 2992 pixels
Battery capacity	3830 mAh	3950 mAh
Battery type	LiPo 3S	LiPo 4S
Voltage	11.4 V	15.4 V
Overall flight time	60 min (Thingvallavatn)	15 min (Ellidavatn)
