# Supplementary material for: Remote sensing of salmonid spawning sites in freshwater ecosystems: The potential of low-cost UAV data
Source: PLoS One. 2023 Aug 29;18(8):e0290736. doi: 10.1371/journal.pone.0290736 (PMC10464957; doi:10.1371/journal.pone.0290736)
Supplement: S2 Table — Accuracy assessment of the seven supervised image classification methods of the spawning redd class in lake Thingvallavatn. Producer’s Accuracy (PA) and User’s Accuracy (UA) are presented in percentages and pixels before post-classification methods have been applied. Table furthermore reports overall accuracy (%) and the kappa coefficient (k). (PDF) [file pone.0290736.s002.pdf]

S2 Table. Accuracy assessment spawning redd class in lake Thingvallavatn. Accuracy assessment of the seven supervised image classification methods of the spawning redd class in lake Thingvallavatn. Producer's Accuracy (PA) and User's Accuracy (UA) are presented in percentages and pixels before post-classification methods have been applied. Table furthermore reports overall accuracy (%) and the kappa coefficient (k).

	Spawning redds class					
**Method**	**PA (%)**	**UA (%)**	**PA (pixels)**	**UA (pixels)**	**Overall accuracy (%)**	**κ**
Mahalanobis distance classification	79.70	77.18	805/1010	805/1043	79.89	0.76
Maximum likelihood classification	89.70	90.51	906/1010	906/1001	87.44	0.85
Minimum distance classification	68.71	57.40	694/1010	694/1209	65.93	0.59
Neural net classification	91.09	79.65	920/1010	920/1155	83.15	0.80
Parallelepiped classification	57.33	76.08	579/1010	579/761	46.38	0.38
Spectral angle mapper classification	54.16	66.63	547/1010	547/821	75.14	0.70
Spectral information divergence classification	42.48	35.11	429/1010	429/1222	29.55	0.15
