# Supplementary material for: Remote sensing of salmonid spawning sites in freshwater ecosystems: The potential of low-cost UAV data
Source: PLoS One. 2023 Aug 29;18(8):e0290736. doi: 10.1371/journal.pone.0290736 (PMC10464957; doi:10.1371/journal.pone.0290736)
Supplement: S3 Table — Results of accuracy assessment of the maximum likelihood classification algorithm in lake Thingvallavatn before and after applying post-classification methods. Reported are producer’s Accuracy (PA) and User’s Accuracy (UA) by class. (PDF) [file pone.0290736.s003.pdf]

S3 Table. Accuracy assessment maximum likelihood lake Thingvallavatn. Results of accuracy assessment of the maximum likelihood classification algorithm in lake Thingvallavatn before and after applying post-classification methods. Reported are producer's Accuracy (PA) and User's Accuracy (UA) by class.

	Before post-classification methods		After post-classification methods	
**Class**	**PA (%)**	**UA (%)**	**PA (%)**	**UA (%)**
Spawning redds	89.70	90.51	90.99	96.33
Vegetation	83.12	85.67	86.10	88.83
Underwater rocks	85.88	73.23	91.21	78.77
Deep water	90.41	90.14	96.50	90.79
Shoreline	96.56	94.33	99.41	95.92
Surface rocks	79.71	93.38	81.42	96.49
Overall accuracy (%)	87.44		90.78	
Kappa coefficient	0.85		0.89	
