# Supplementary material for: Remote sensing of salmonid spawning sites in freshwater ecosystems: The potential of low-cost UAV data
Source: PLoS One. 2023 Aug 29;18(8):e0290736. doi: 10.1371/journal.pone.0290736 (PMC10464957; doi:10.1371/journal.pone.0290736)
Supplement: S4 Table — Results of accuracy assessment of the neural network classification algorithm in lake Thingvallavatn before and after applying post-classification methods. Reported are producer’s Accuracy (PA) and User’s Accuracy (UA) by class. (PDF) [file pone.0290736.s004.pdf]

S4 Table. Accuracy assessment neural net lake Thingvallavatn. Results of accuracy assessment of the neural network classification algorithm in lake Thingvallavatn before and after applying post-classification methods. Reported are producer's Accuracy (PA) and User's Accuracy (UA) by class.

	Before post-classification methods		After post-classification methods	
**Class**	**PA (%)**	**UA (%)**	**PA (%)**	**UA (%)**
Spawning redds	91.09	79.65	92.18	88.92
Vegetation	67.73	85.89	70.31	92.07
Underwater rocks	86.08	66.31	89.73	69.13
Deep water	82.72	92.10	90.91	92.11
Shoreline	94.49	94.22	98.92	95.00
Surface rocks	77.29	87.95	80.25	90.85
Overall accuracy (%)	83.15		86.95	
Kappa coefficient	0.80		0.84	
