# Supplementary material for: Remote sensing of salmonid spawning sites in freshwater ecosystems: The potential of low-cost UAV data
Source: PLoS One. 2023 Aug 29;18(8):e0290736. doi: 10.1371/journal.pone.0290736 (PMC10464957; doi:10.1371/journal.pone.0290736)
Supplement: S5 Table — Results of accuracy assessment of the maximum likelihood classification algorithm in lake Ellidavatn before and after applying post-classification methods. Reported are producer’s Accuracy (PA) and User’s Accuracy (UA) by class. (PDF) [file pone.0290736.s005.pdf]

S5 Table. Accuracy assessment maximum likelihood lake Ellidavatn. Results of accuracy assessment of the maximum likelihood classification algorithm in lake Ellidavatn before and after applying post-classification methods. Reported are producer's Accuracy (PA) and User's Accuracy (UA) by class.

	Before post-classification methods		After post-classification methods	
**Class**	**PA (%)**	**UA (%)**	**PA (%)**	**UA (%)**
Spawning redds	91.79	84.67	95.15	87.06
Vegetation	88.75	83.24	91.31	85.10
Underwater rocks	75.47	96.85	75.22	99.46
Aquatic vegetation	88.26	91.76	90.68	95.21
Anthropogenic feature	93.20	91.97	95.88	92.77
Sediment	85.97	75.70	87.65	77.18
Overall accuracy (%)	86.88		88.89	
Kappa coefficient	0.84		0.87	
