# Supplementary material for: Remote sensing of salmonid spawning sites in freshwater ecosystems: The potential of low-cost UAV data
Source: PLoS One. 2023 Aug 29;18(8):e0290736. doi: 10.1371/journal.pone.0290736 (PMC10464957; doi:10.1371/journal.pone.0290736)
Supplement: S6 Table — Results of accuracy assessment of the neural network classification algorithm in lake Ellidavatn before and after applying post-classification methods. Reported are producer’s Accuracy (PA) and User’s Accuracy (UA) by class. (PDF) [file pone.0290736.s006.pdf]

S6 Table. Accuracy assessment neural net lake Ellidavatn. Results of accuracy assessment of the neural network classification algorithm in lake Ellidavatn before and after applying post-classification methods. Reported are producer's Accuracy (PA) and User's Accuracy (UA) by class.

	Before post-classification methods		After post-classification methods	
**Class**	**PA (%)**	**UA (%)**	**PA (%)**	**UA (%)**
Spawning redds	96.64	82.03	99.80	85.58
Vegetation	90.42	68.87	91.71	69.85
Underwater rocks	78.00	92.89	77.11	95.74
Aquatic vegetation	55.36	83.90	57.69	87.68
Anthropogenic feature	91.00	89.79	94.64	91.91
Sediment	65.11	62.20	65.58	60.54
Overall accuracy (%)	79.08		80.66	
Kappa coefficient	0.75		0.77	
