# Supplementary material for: Remote sensing of salmonid spawning sites in freshwater ecosystems: The potential of low-cost UAV data
Source: PLoS One. 2023 Aug 29;18(8):e0290736. doi: 10.1371/journal.pone.0290736 (PMC10464957; doi:10.1371/journal.pone.0290736)
Supplement: Attachment — Submitted filename: Response to Reviewers.docx [file pone.0290736.s007.docx]

**View Letter**

| Date: | Jun 22 2023 06:38PM |
| --- | --- |
| To: | "Lieke Ponsioen" lieke@hi.is |
| From: | "PLOS ONE" plosone@plos.org |
| Subject: | PLOS ONE Decision: Revision required [PONE-D-23-17918] |

PONE-D-23-17918

Remote sensing of salmonid spawning sites in freshwater ecosystems: The potential of low-cost UAV data

PLOS ONE

Dear Dr. Ponsioen,

Thank you for submitting your manuscript to PLOS ONE. After careful consideration, we feel that it has merit but does not fully meet PLOS ONE’s publication criteria as it currently stands. Therefore, we invite you to submit a revised version of the manuscript that addresses the points raised during the review process.

Please submit your revised manuscript by Aug 06 2023 11:59PM. If you will need more time than this to complete your revisions, please reply to this message or contact the journal office at [plosone@plos.org](mailto:plosone@plos.org). When you're ready to submit your revision, log on to https://www.editorialmanager.com/pone/ and select the 'Submissions Needing Revision' folder to locate your manuscript file.

Please include the following items when submitting your revised manuscript:

A rebuttal letter that responds to each point raised by the academic editor and reviewer(s). You should upload this letter as a separate file labeled 'Response to Reviewers'.

A marked-up copy of your manuscript that highlights changes made to the original version. You should upload this as a separate file labeled 'Revised Manuscript with Track Changes'.

An unmarked version of your revised paper without tracked changes. You should upload this as a separate file labeled 'Manuscript'.

If you would like to make changes to your financial disclosure, please include your updated statement in your cover letter. Guidelines for resubmitting your figure files are available below the reviewer comments at the end of this letter.

If applicable, we recommend that you deposit your laboratory protocols in protocols.io to enhance the reproducibility of your results. Protocols.io assigns your protocol its own identifier (DOI) so that it can be cited independently in the future. For instructions see: <https://journals.plos.org/plosone/s/submission-guidelines#loc-laboratory-protocols>. Additionally, PLOS ONE offers an option for publishing peer-reviewed Lab Protocol articles, which describe protocols hosted on protocols.io. Read more information on sharing protocols at <https://plos.org/protocols?utm_medium=editorial-email&utm_source=authorletters&utm_campaign=protocols>.

We look forward to receiving your revised manuscript.

Kind regards,

Benigno Elvira, Ph.D.
Academic Editor
PLOS ONE

Journal Requirements:

When submitting your revision, we need you to address these additional requirements.

1. Please ensure that your manuscript meets PLOS ONE's style requirements, including those for file naming. The PLOS ONE style templates can be found at 
https://journals.plos.org/plosone/s/file?id=wjVg/PLOSOne_formatting_sample_main_body.pdf and 
https://journals.plos.org/plosone/s/file?id=ba62/PLOSOne_formatting_sample_title_authors_affiliations.pdf

2. Please note that PLOS ONE has specific guidelines on code sharing for submissions in which author-generated code underpins the findings in the manuscript. In these cases, all author-generated code must be made available without restrictions upon publication of the work. Please review our guidelines at https://journals.plos.org/plosone/s/materials-and-software-sharing#loc-sharing-code and ensure that your code is shared in a way that follows best practice and facilitates reproducibility and reuse.

3. We note that the grant information you provided in the ‘Funding Information’ and ‘Financial Disclosure’ sections do not match.

When you resubmit, please ensure that you provide the correct grant numbers for the awards you received for your study in the ‘Funding Information’ section.

4. In your Data Availability statement, you have not specified where the minimal data set underlying the results described in your manuscript can be found. PLOS defines a study's minimal data set as the underlying data used to reach the conclusions drawn in the manuscript and any additional data required to replicate the reported study findings in their entirety. All PLOS journals require that the minimal data set be made fully available. For more information about our data policy, please see http://journals.plos.org/plosone/s/data-availability.

"Upon re-submitting your revised manuscript, please upload your study’s minimal underlying data set as either Supporting Information files or to a stable, public repository and include the relevant URLs, DOIs, or accession numbers within your revised cover letter. For a list of acceptable repositories, please see http://journals.plos.org/plosone/s/data-availability#loc-recommended-repositories. Any potentially identifying patient information must be fully anonymized.

Important: If there are ethical or legal restrictions to sharing your data publicly, please explain these restrictions in detail. Please see our guidelines for more information on what we consider unacceptable restrictions to publicly sharing data: http://journals.plos.org/plosone/s/data-availability#loc-unacceptable-data-access-restrictions. Note that it is not acceptable for the authors to be the sole named individuals responsible for ensuring data access.

We will update your Data Availability statement to reflect the information you provide in your cover letter.

5. Your ethics statement should only appear in the Methods section of your manuscript. If your ethics statement is written in any section besides the Methods, please move it to the Methods section and delete it from any other section. Please ensure that your ethics statement is included in your manuscript, as the ethics statement entered into the online submission form will not be published alongside your manuscript.

[Note: HTML markup is below. Please do not edit.]

Reviewers' comments:

Reviewer's Responses to Questions

**Comments to the Author**

1. Is the manuscript technically sound, and do the data support the conclusions?

The manuscript must describe a technically sound piece of scientific research with data that supports the conclusions. Experiments must have been conducted rigorously, with appropriate controls, replication, and sample sizes. The conclusions must be drawn appropriately based on the data presented.

Reviewer #1: Yes

Reviewer #2: No

2. Has the statistical analysis been performed appropriately and rigorously?

Reviewer #1: Yes

Reviewer #2: No

3. Have the authors made all data underlying the findings in their manuscript fully available?

The [PLOS Data policy](http://www.plosone.org/static/policies.action#sharing) requires authors to make all data underlying the findings described in their manuscript fully available without restriction, with rare exception (please refer to the Data Availability Statement in the manuscript PDF file). The data should be provided as part of the manuscript or its supporting information, or deposited to a public repository. For example, in addition to summary statistics, the data points behind means, medians and variance measures should be available. If there are restrictions on publicly sharing data—e.g. participant privacy or use of data from a third party—those must be specified.

Reviewer #1: Yes

Reviewer #2: Yes

4. Is the manuscript presented in an intelligible fashion and written in standard English?

PLOS ONE does not copyedit accepted manuscripts, so the language in submitted articles must be clear, correct, and unambiguous. Any typographical or grammatical errors should be corrected at revision, so please note any specific errors here.

Reviewer #1: Yes

Reviewer #2: Yes

5. Review Comments to the Author

Please use the space provided to explain your answers to the questions above. You may also include additional comments for the author, including concerns about dual publication, research ethics, or publication ethics. (Please upload your review as an attachment if it exceeds 20,000 characters)

Reviewer #1: The authors propose an easy low-cost approach to map entire spawning habitats using drones. I believe that this technic is useful for this kind of analysis, but have some limitations.
I believe that study is adequate, but I have some doubts about the validation methodology. I believe that it is necessary more detail about the determination of the extent of the spawning grounds. Why do you need to take embryos? How many embryos? What about scuba diving and video recording? Time effort and efficiency? Please, clarify it.
Finally, I believe that more discussion about the weaknesses and limitations of this technics is necessary. There are a lot of limitations to the use of drones to check fish spawning areas (water turbidity, similar and confusing images, drone accessibility, fish ecological behaviour, etc.) and this is worth to be discussed.

Thank you very much for your feedback and helpful comments.

Confusion has arisen from the validation part of the text due to our poor wording. We have now adjusted our wording to make the steps clearer (115-119). We have taken out the mention of SCUBA diving as we did this to be able to collect the embryos which was done for another unrelated project. One of the steps is to note the presence of eggs to confirm successful spawning, but this can be done by a wide variety of ways, including snorkelling and SCUBA diving but more efficiently by wading through water using boots or waders. Even with SCUBA diving the validating of the spawning grounds took us about an hour.

We have added a paragraph to the discussion to address the limitations of using a drone to map spawning redds (372-391).

Finally, please, include the sentence “The data that support the findings of this study is available on the GeoVis Lab website at https://geovis.hi.is/research/data/” in the text, with the aim to facilitate access.

We have moved our data to a GitHub repository to make it more accessible and have added the sentence in results section (228-229).

Line 19: Why “Although”? please, delete it.

We removed it.

Line 88: please, include the name and year of description of species: e.g. Salmo trutta (Linnaeus, 1758)

This information has been added (91).

Line 91: why i.e.? this is a Latin abbreviation that minds something like “this is”. Please, delete it.

Removed.

I believe that Table 1 is unnecessary. It offers information that can be included in the text.

We have removed the table from the manuscript and any relevant information is mentioned within the text (104-108).

Figures 3 and 4 can be mixed in a unique figure, this facilitates the comparison between images. The same to 5 and 6.

We have done so.

Reviewer #2: I read with interest the work by Ponsioen et al. entitled: "Remote sensing of salmonid spawning sites in freshwater ecosystems: The potential of low-cost UAV data". This paper uses lightweight unmanned aerial vehicles (UAVs) to monitor and map salmon spawning redd, avoiding time-consuming surveys by trained personnel. The work's rationale is interesting and useful in riverine ecology; however, the paper suffers several drawbacks that must be addressed before publication. The material and methods section lacks details to make this study applicable in other environments.

The results only encompass the description of confusion matrices without addressing ecological or methodological aspects.
In my opinion, the paper is not suitable for publication in its current form. However, I recommend that the authors resubmit a new, improved version after addressing significant issues.
We thank you for your thoughtful and detailed comments.

To show how our method can be applied to other environments we have adjusted the pipeline figure (Fig 4) and we have added the information to the conclusion (425-449).

We have moved methodological aspects to the results (218-255), this includes the selection of the drone flight altitude and the seven tested algorithms. We agree that adding ecological aspects is an important aspect, but we decided to focus on the spawning redds in this paper and selected the other endmember classes to be able to distinguish the spawning redds. To address ecological aspects further enhancement of the analyses is necessary and while possible, we believe it would bring a lengthy discussion and water down the main message we are trying to convey that it is an easy to use method to detect and map spawning redds. Instead, we have added in the discussion a sentence of the scope to classify other endmember classes in such a way that ecological analyses can be done (417-422).

Specific comments
Introduction:
I suggest starting with the role of salmonids, stressing ecological aspects and their role as fisheries resources. Moreover, discussing the nursery role of specific habitats for setting redds, I suggest talking about how nurseries are defined and measured (See Beck et al., 2001, 2003). Considering other papers dealing with riverine mapping, the paragraph regarding using UAV-based imagery should be better developed. Since you are proposing a new approach capable of lowering the effort required for direct in situ sampling, I suggest also focusing on the other limitation (other than time) of traditional visual census methods, such as inter-observe variability, limited area and safety. Moreover, the superimposition issue should be better explained and discussed to highlight. Direct observation remains better for solving this issue than aerial imagery; therefore, this aspect should be clarified.

Thank you, we have now changed the introduction to reflect your suggestions (34-47).

We have also added a line in the discussion (355-359) to clarify the superimposition.

Lines 68-69: What do you mean by estimating "the entirely of spawning habitats"? The paper lacks any reference regarding such habitats' geographic extension and cover. In the results section, I suggest comparing the two surveyed sites considering the extension of these mapped habitats.

We have revised the text and made it more clear (77-79).

As you point out in another comment the two spawning sites differ significantly in redd cover and redds differ in size, complexity and extent as well. So while the spawning grounds of lake Ellidavatn are not as complex and a single image covers all the redds, the spawning grounds in lake Thingvallavatn are highly complex and a composite image is needed to be able to detect all the redds. Therefore, we chose to use a single image for both of them to keep our results consistent.

Line 72: Several times, you highlight that such monitoring can be done without using trained personnel usually working in the field. However, please consider the time and the trained operators needed for deploying and flying the drone other than the skill required for data processing and analysis.

We have added this to the discussion along with other weaknesses of using drones to map spawning redds (372-391). Some skill is indeed necessary but with time drones are getting more user friendly. New features are constantly being added such as GPS and collision avoidance technology. In our study we did use an experienced drone pilot to collect the data, but drones are constantly evolving making it nowadays possible for amateurs to use them. Every person on our team has now successfully collected data following our own pipeline.

Material and methods
The study areas are very well described; however, many of these characteristics are not essential in applying this method, so I suggest streamlining this part by moving, for instance, the description of species in the introduction. I also recommend adding an image of a redd taken from the ground, maybe underwater, to improve Fig. 1d.

We have streamlined the study area subsection (84-108).

Following your recommendation we looked at different redd images taken underwater (see an example below). Neither of the underwater images examined showed a redd well enough. Thus, we decided to keep our current figure (fig 1d).

Lines 126-130: The workflow in this section regarding the study area seems out of topic. It should be improved by adding more information in each part and inserting it at the end of the material and methods section to summarize all operative steps.

We have removed a large part from the study area subsection (84-108). Furthermore, the pipeline is moved to the results (217), and following one of your previous comments the pipeline has been adjusted (Fig 4).

Data acquisition
Line 136: You mentioned that SCUBA diving had been carried out as part of the ground truthing, but in the paper, this part of data validation is missing, and all the confusion matrices have been built using only imagery interpretation. Why did you not use such information collected directly on the field as validation points for your classification?

Fair comment, this was also noticed by the other reviewer. Confusion has arisen from the validation part of the text due to our poor wording. We have now adjusted our wording to make the steps clearer (115-119). We have taken out the mention of SCUBA diving as we did this to be able to collect the embryos which was done for another unrelated project. One of the steps is to note the presence of eggs to confirm successful spawning, but this can be done by a wide variety of ways, including snorkelling and SCUBA diving but more efficiently by wading through water using boots or waders. Even with SCUBA diving the validating of the spawning grounds took us about an hour.

We also added (125-127) that the ground truthing was done on the day of data acquisition by visually confirming the presence of redds from the shore. We hope this clears it up.

Lines 138-141: Two different drones with different sensors (sensors size, resolution, ISO sensitivity, etc.) have been used for imagery acquisition, so it could be useful to discuss the best camera settings and accuracies of the results, also considering the quality of the acquired photo.

We have added this to the discussion (386-391).

Line 156-157: How did you stitch together more than one image collected in a study area?

We have clarified the text stating that a singular image was used (137-138).

Results:
After discussing the best classification algorithm, I suggest adding some methodological and ecologic value results. Which is the best drone for such kind of acquisition and why? Camera settings and flight altitude are important for imagery acquisition, so some results should address these aspects. The two areas differ significantly in redds cover? If yes, why?

We have moved a large part of our material and methods section to the results (218-255) as this encompassed methodological results (such as flight altitude selection and algorithm selection). Furthermore, we have added information about the camera settings to the material and methods (121-124) and discuss it in the discussion (386-391).

And indeed the two spawning grounds differ in redds cover. This is due to lake Thingvallavatn having one major spawning ground and a short spawning time of two months while in lake Ellidavatn there are more locations with spawning grounds as well as a longer spawning time (September to the end of November).

Is it unclear if a photogrammetric approach is used to generate the photomosaic of the first area. If so, how have the other cartographic products (DSMs) been validated? Can they serve to improve habitat classification? If you have not applied any kind of Structure from Motion (SfM) processing, the imagery used can be affected by significant errors in positioning. Regarding these last aspects, all the generated maps cannot be used as cartographic products being lacking in georeferencing (grids with corresponding coordinates) and orientation (north arrows).

In our study, the focus was not on generating a photomosaic using a photogrammetric approach. Therefore, no photogrammetric techniques were employed for this purpose since we worked with a single UAV image in the analyses. We apologise if this was not clearly conveyed in the article. The absence of a photomosaic was intentional as it fell outside the scope of our study objectives. The validation process involved rigorous cross-referencing with ground observations made during data collection at the particular location that was used in the analysis.

To clarify, we have added a paragraph where we acknowledge the needs for and benefits of using photogrammetric techniques as well as the importance of georeferencing for long term monitoring purposes (409-414).

Discussion
In the discussion section, some important considerations regarding the use of this data for population monitoring should be added. Open-source software can be used, but in your work, the pipeline proposed is based on ENVI software. The same results could be achieved also with QGIS? Could limitations exist?

We have added some more information on QGIS as this is a very suitable platform to which our pipeline can be transferred (405-407). A Semi-automated classification plugin has been developed for QGIS which is able to handle our pipeline.

6. PLOS authors have the option to publish the peer review history of their article ([what does this mean?](https://journals.plos.org/plosone/s/editorial-and-peer-review-process#loc-peer-review-history)). If published, this will include your full peer review and any attached files.


If you choose “no”, your identity will remain anonymous but your review may still be made public.


**Do you want your identity to be public for this peer review?** For information about this choice, including consent withdrawal, please see our [Privacy Policy](https://www.plos.org/privacy-policy).

Reviewer #1: No

Reviewer #2: No

[NOTE: If reviewer comments were submitted as an attachment file, they will be attached to this email and accessible via the submission site. Please log into your account, locate the manuscript record, and check for the action link "View Attachments". If this link does not appear, there are no attachment files.]

While revising your submission, please upload your figure files to the Preflight Analysis and Conversion Engine (PACE) digital diagnostic tool, <https://pacev2.apexcovantage.com/>. PACE helps ensure that figures meet PLOS requirements. To use PACE, you must first register as a user. Registration is free. Then, login and navigate to the UPLOAD tab, where you will find detailed instructions on how to use the tool. If you encounter any issues or have any questions when using PACE, please email PLOS at [figures@plos.org](mailto:figures@plos.org). Please note that Supporting Information files do not need this step.

*In compliance with data protection regulations, you may request that we remove your personal registration details at any time.* [*(Remove my information/details)*](https://www.editorialmanager.com/pone/login.asp?a=r)*. Please contact the publication office if you have any questions.*
